# Variation in Responses of Fishes across Multiple Reserves within a Network of Marine Protected Areas in Temperate Waters

**DOI:** 10.1371/journal.pone.0118502

**Published:** 2015-03-11

**Authors:** Richard M. Starr, Dean E. Wendt, Cheryl L. Barnes, Corina I. Marks, Dan Malone, Grant Waltz, Katherine T. Schmidt, Jennifer Chiu, Andrea L. Launer, Nathan C. Hall, Noëlle Yochum

**Affiliations:** 1 California Sea Grant Extension Program, Moss Landing Marine Laboratories, Moss Landing, California, United States of America; 2 Moss Landing Marine Laboratories, Moss Landing, California, United States of America; 3 California Polytechnic University, San Luis Obispo, California, United States of America; 4 University of California Santa Cruz, Santa Cruz, California, United States of America; 5 Oregon State University, Corvallis, Oregon, United States of America; Bangor University, UNITED KINGDOM

## Abstract

Meta-analyses of field studies have shown that biomass, density, species richness, and size of organisms protected by no-take marine reserves generally increase over time. The magnitude and timing of changes in these response variables, however, vary greatly and depend upon the taxonomic groups protected, size and type of reserve, oceanographic regime, and time since the reserve was implemented. We conducted collaborative, fishery-independent surveys of fishes for seven years in and near newly created marine protected areas (MPAs) in central California, USA. Results showed that initially most MPAs contained more and larger fishes than associated reference sites, likely due to differences in habitat quality. The differences between MPAs and reference sites did not greatly change over the seven years of our study, indicating that reserve benefits will be slow to accumulate in California’s temperate eastern boundary current. Fishes in an older reserve that has been closed to fishing since 1973, however, were significantly more abundant and larger than those in associated reference sites. This indicates that reserve benefits are likely to accrue in the California Current ecosystem, but that 20 years or more may be needed to detect significant changes in response variables that are due to MPA implementation. Because of the high spatial and temporal variability of fish recruitment patterns, long-term monitoring is needed to identify positive responses of fishes to protection in the diverse set of habitats in a dynamic eastern boundary current. Qualitative estimates of response variables, such as would be obtained from an expert opinion process, are unlikely to provide an accurate description of MPA performance. Similarly, using one species or one MPA as an indicator is unlikely to provide sufficient resolution to accurately describe the performance of multiple MPAs.

## Introduction

Marine protected areas (MPAs) in general, and no-take marine reserves specifically, are increasingly being used as tools for fisheries management and conservation of marine resources around the world [1], [2]. Empirical evidence for the ecological success of marine reserves is accumulating and meta-analyses of field studies show that marine reserves typically yield positive results with respect to an increase in the response variables of biomass, density, species richness, and size of organisms protected (e.g., [3–6]). The magnitude and timing of changes in response variables across the world, however, varies greatly and depends upon the taxonomic groups protected, life history of species protected, size of reserve, protection level and amount of enforcement, oceanographic regime, and time since the reserve was implemented [6], [7]. This variability may be one reason why the debate continues about the value of marine reserves, especially in temperate environments [6], and provides strong rationale that monitoring is critical for the evaluation of reserves as an approach to managing marine resources [8].

Over the last ten years, the state government of California, USA, has implemented a long-planned network of MPAs. After two unsuccessful efforts to establish MPAs, a comprehensive planning process was undertaken to design networks of MPAs that were based on size and spacing guidelines designed to ensure connectivity among MPAs [9], [10]. The first set of these networks was established in central California in 2007; it contains 29 MPAs and protects a total of 529 km^2^ (17.9%) of nearshore habitats, with no-take marine reserves protecting 218 km^2^ (7.4%) of the state’s central coast waters [11]. The theoretical benefits of the network of MPAs include providing habitat (and therefore species) diversity and redundancy, protection against localized environmental catastrophe and climate change, maintenance of genetic diversity, population persistence, and distribution of costs and benefits with respect to fisheries.

The state law that led to the formation of the new MPAs contained six specific goals [11]. Two of these goals related to the long-term benefits that are intended to accrue from increased resource protection are: 1) to protect the natural diversity and abundance of marine life and the structure, function, and integrity of marine ecosystems, and 2) to help sustain, conserve, and protect marine life populations, including those of economic value, and rebuild those that are depleted. An additional long-term goal is: 3) to ensure that the state’s MPAs are designed and managed, to the extent possible, as a network. The other three goals relate to recreation, education, intrinsic social values, and governance issues. Inherent in the development of the goals was the expectation to monitor and adaptively manage MPAs.

The state of California is now developing metrics that can be used to monitor, evaluate, and adaptively manage MPAs with respect to those predicted benefits. The focus of the three adopted goals relating to marine populations is on maintenance of biodiversity and population persistence. Monitoring projects that provide data for adaptive management need be designed to evaluate changes in response variables such as species diversity, population density, biomass, age structure, and larval production. Although the level of detail needed to adaptively manage MPAs depends upon the spatial scale of the management action (e.g., at a broad level such as adding or deleting MPAs or at a fine scale such as changing boundaries of an existing MPA), at the root of adaptive management is the need to track changes in metrics over time. An effective adaptive management process thus requires a well-designed monitoring program with a statistically rigorous sampling design.

In 2006, we formed an alliance of academic and agency scientists, members of the fishing community, and non-governmental organizations to address the need for baseline data and continued monitoring of MPAs. This group, the California Collaborative Fisheries Research Program (CCFRP), adopted sampling protocols designed to monitor and evaluate the effectiveness of marine reserves, primarily with respect to nearshore fishes [12], [13]. In the summer and fall from 2007–2013 we worked with 12 Commercial Passenger Fishing Vessels (charter fishing vessels for hire) and > 700 different volunteer anglers to monitor four MPAs and co-located reference sites in central California. Here we describe the approach we took to evaluate differences in initial conditions of central California MPAs, factors influencing the variability among response variables in central California marine reserves, and changes in response variables across multiple MPAs. We did not attempt to evaluate larval connectivity of fish populations among MPAs as metrics and effective techniques to test the efficacy of networks are still being developed. Instead, we addressed a series of questions related to the predicted responses of fish populations to marine reserve establishment:

1. Were the initial conditions in individual MPAs and their co-located reference sites (REF) similar with respect to species composition, catch rates, biomass, and size-frequency distributions?
2. Were there changes in species composition, catch rates, biomass, and size-frequency distributions during the first seven years of MPA designation?
3. Did all populations respond similarly across the multiple MPAs we studied, such that one species or MPA could be used as an indicator of responses for MPAs in other areas?

## Methods

We incorporated local knowledge and expertise into a fishery-independent sampling design that was adopted after a series of workshops with fishermen, harbor officials, and scientists from agency, academic, and conservation organizations [12], [13]. The protocols we developed for monitoring MPAs were based on a stratified random sampling design wherein we used seafloor maps and fishermen’s knowledge to stratify the sampling areas by habitat for nearshore rockfishes (*Sebastes* spp.) and then sampled those areas by conducting standardized hook-and-line fishing surveys (see [13] for more detail about methods). REF sites for each MPA were selected based on their proximity, similarity in depth, bathymetry, substrate characteristics, and oceanographic conditions found within MPAs. All REF sites were from 0.5–10 km away from the corresponding MPAs. We intentionally chose REF sites close to MPAs because we believed that environmental variation among different areas would be a greater source of difference than any spillover that might occur immediately following MPA designation.

### Study areas

**Fig 1:**
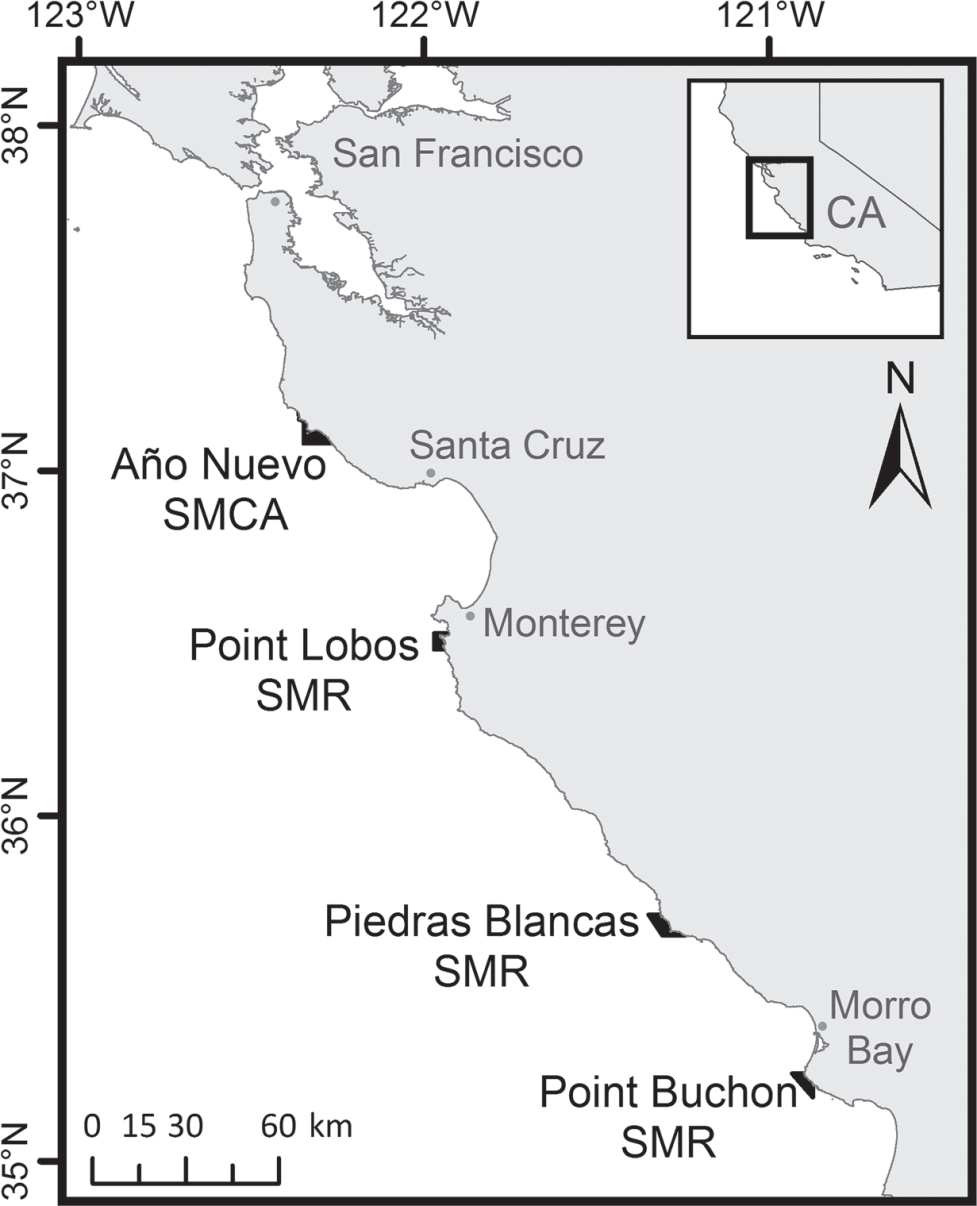
Location of the four study areas in central California. Año Nuevo State Marine Conservation Area (SMCA), and the Point Lobos, Piedras Blancas, and Point Buchon State Marine Reserves (SMRs).

Sampling occurred in four different geographic areas along 350 km of coastline. Each area contained a MPA and REF site. Within each site multiple grid cells were established and served as sample units. Sampling areas included three State Marine Reserves that are closed to all fishing (Point Lobos, Piedras Blancas, and Point Buchon), one State Marine Conservation Area that is closed to all fishing (Año Nuevo) but allows harvest of giant kelp (*Macrocystis pyrifera*) by hand, and the REF sites associated with each of these MPAs (Fig. 1). The Año Nuevo, Point Lobos, Piedras Blancas, and Point Buchon MPAs encompass areas of 26.4 km^2^, 14.0 km^2^, 26.9 km^2^, and 17.4 km^2^, respectively. Within the boundaries of each MPA and REF site, 500 m by 500 m grid cells were delineated in rocky habitats in water < 40 m deep (to limit fishing mortality associated with barotrauma). A total of 22 grid cells in Año Nuevo, 17 cells in Point Lobos, 57 cells in Piedras Blancas, and 22 cells in Point Buchon were designated, numbered, and then chosen at random to be sampled on a given day. Our work in these MPAs was approved by the California Department of Fish and Wildlife (CDFW) as part of CDFW scientific collecting permits #2613 and #6681. Also, we obtained permission from the California Department of Parks and Recreation to sample in the marine reserve in the Point Lobos State Park.

### Sampling protocols and ethics statement

Surveys were conducted annually in the four areas from 2007–2013 (except that surveys in the Piedras Blancas area began in 2008). Surveys occurred in the late summer period from mid–July through September when ocean conditions in the region are most consistent. Each MPA or REF site was sampled two days a month (usually within a few days of each other) for two months per year. Before each day of fishing, four of the grid cells in a given MPA or REF site were randomly chosen for sampling. Volunteer anglers were recruited from various fishing clubs, online fishing websites, and from previous collaborative studies. We used a standardized mixture of fishing gear (metal jigs, feathered lures, and barbless baited hooks) in order to capture a variety of species and cover the spectrum of typical hook-and-line fishing gear used in this region [13].

Captured fishes were identified to species, measured, tagged with a T-bar anchor tag (unless the fish was in poor condition or was too small to tag), and released. Lengths reported are total length, defined as the distance from the tip of the snout to the most posterior part of the caudal fin without compressing the tail. We recorded the locations (latitude and longitude) and depths where fishes were released. The effects of barotrauma were reduced with venting needles and descending devices, and by minimizing the duration of time that the fishes were on board the vessel. We aimed to process and release fish in < 5 min in order to minimize effects of barotrauma and handling stress. The San Jose State University and the California Polytechnic State University Institutional Animal Care and Use Committee (IACUC) approved this study as San Jose State University IACUC Animal Protocol number 824 and Cal Poly IACUC Animal Protocol number 1205.

### Response variables

**Fig 2:**
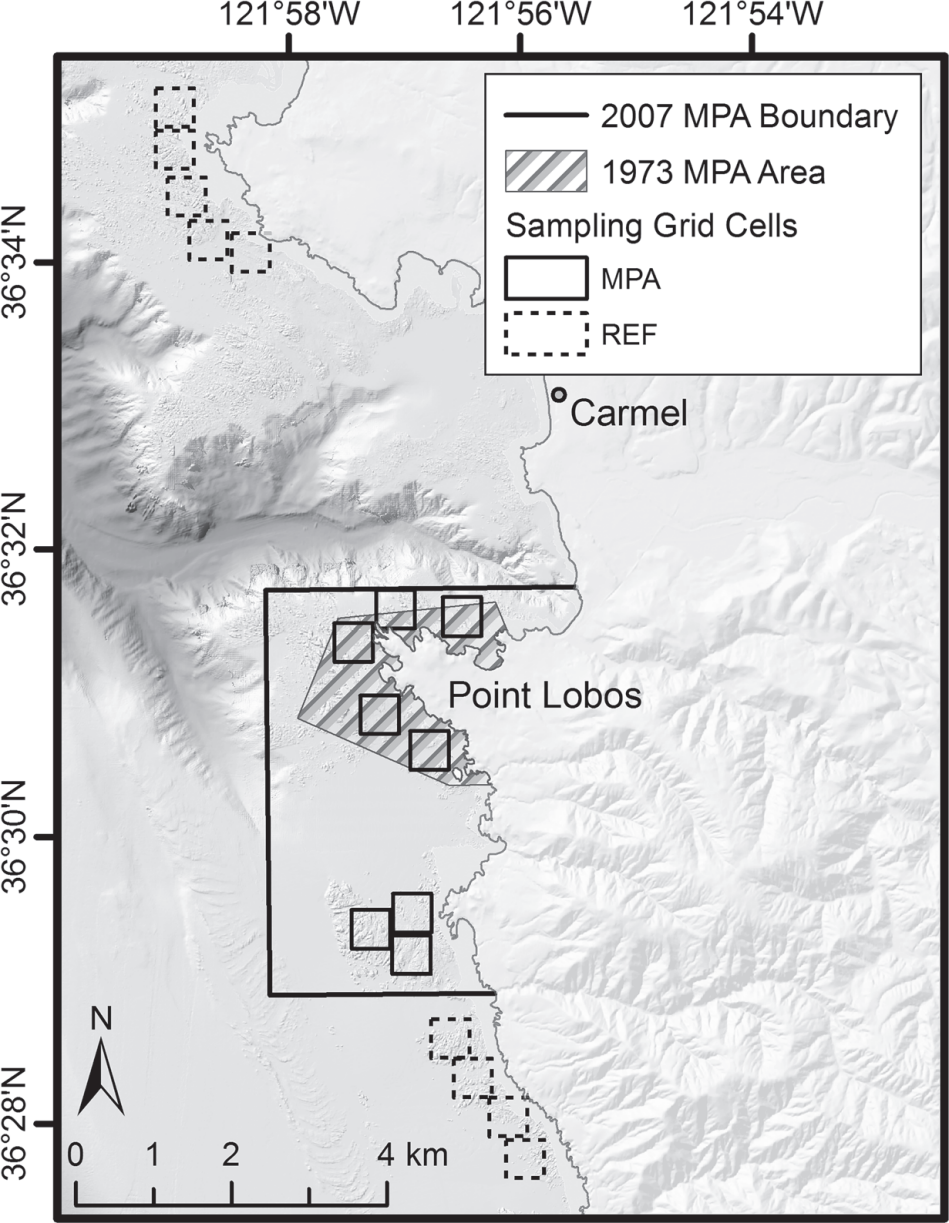
Locations of the sampling in the Point Lobos area. Grid cells, 500 m by 500 m in size, were established as sampling locations in both the Point Lobos marine protected area (MPA) and reference (REF) site. The area designated by diagonal lines around Point Lobos has been closed to all fishing since 1973.

Response variables considered in this study include catch-per-angler-hour (CPUE), biomass caught-per-angler-hour (BPUE), and the mean lengths of fishes caught during a sampling cell visit. Catch rates are reported as the annual mean CPUE of fishing with two hooks per fishing rod and were calculated by dividing the total number of fishes caught by total angler hours of fishing in a sampling cell in a day. BPUE was calculated for each fish caught by converting lengths to weights for each fish using published length-weight relationships for each species. Weights were then used to calculate the total BPUE during each visit to a sampling cell. CPUE and BPUE for each grid cell in each day were averaged over each year to estimate trends in CPUE and BPUE over time in MPA and REF sites. CPUE and BPUE in the 2007 and 2008 sample years were deemed to be starting conditions, except for the pre-existing reserve portion of Point Lobos MPA that was treated as distinct from the newly designated MPA area because fishing has been prohibited there since 1973 (Fig. 2, labeled “1973 MPA Area”). Estimates of mean lengths, CPUE, and BPUE were generated for all species caught, but analyses presented here focus on the eleven most abundant species, those that each represent > 1% of the total catch for all areas combined.

### Geographic comparison of species composition

Unpublished maps of the rock lithology and associated habitats in central California (http://walrus.wr.usgs.gov/mapping/csmp/, Accessed 5 January 2015) led us to expect that species compositions should be more similar between a given MPA and REF than among other areas along the coast. To test this, we conducted a multivariate comparison of species composition among areas and between MPA and REF sites. We did this by comparing the similarities between the relative species compositions among areas and sites using cluster analysis and non-metric multi-dimensional scaling (MDS) plots based on Bray-Curtis similarity indices (PRIMER v.6). In addition to the graphical analyses, we compared differences in species compositions among areas with a permutation MANOVA, using the “Vegan” package in the “R” statistical program.

### Mixed-model repeated-measures ANOVA

We used a mixed-model, repeated-measures analysis of variance (ANOVA) to test for differences in species abundance and mean sizes of fishes inside and outside of MPAs, both at the time of implementation, and as change over time using data from the entire seven-year sampling period (SAS v9.4). Main effects in the model included area (the four MPA areas surveyed), site (MPA or REF) and sample year as a continuous covariate. A day of sampling in a grid cell was used as the sample unit. Models were run individually for each of the 11 most abundant species recorded, and with all of those species combined. Response variables were evaluated for normality using probability distribution plots prior to analysis, leading us to apply a square root transformation of CPUE and BPUE. Analyses were conducted for each area individually and for all areas together as an analysis of network-wide MPA effects. Also, we conducted a factor analysis in SPSS to identify groups of species with highly correlated distributions, then used those groups to determine how well CPUE obtained from one species could predict relative abundance of co-occurring species.

### Multiple-regression analysis using environmental variables

To assess the amount of variance in the BPUE response variable that could be accounted for by spatial (sample cell, site, area) or temporal (sample year) factors relative to environmental conditions at the time of sampling, we conducted a multiple regression analysis using forward model selection and calculated variation accounted for by each variable (semi-partial eta-square). Environmental variables used in this study as potential correlates to individual species’ catch rates included *in situ* recordings of sea-surface temperature and wind-speed, depth and bottom relief measurements recorded from ship-board echo sounders, observed numbers of harbor seals (*Phoca vitulina*) and California sea lions (*Zalophus californianus*) in the area at the time of fishing, buoy-derived estimates of wave energy, winds, and temperature calculated using 3, 24, 48, and 72 hour moving averages, and estimates of depth and relief derived from multibeam sonar data collected and processed by the California Seafloor Mapping Project [14]. Seafloor digital elevation models of the MPA and REF sites generated at 2 m resolution were used to calculate the mean, range, and standard deviation of three types of seafloor characteristics: depth, slope, and rugosity. Mean estimates of each of these bathymetric variables were calculated from the area within each sampling cell. All environmental variables used in model selection were first tested for colinearity (variance inflation factor < 3).

## Results

We conducted 244 days of hook-and-line fishing surveys inside MPAs and in associated REF sites between 2007 and 2013. A total of 717 different volunteer anglers provided 6,813 hours of timed fishing over the entire study period. With their help, we caught and released 46,853 fishes, of which 33,418 were tagged. We caught a total of 43 species (S1 Table), however, 11 species comprised 96.1% of the catch (Table 1). In all areas, rockfishes were the predominant species group, comprising 94.1% of the total catch (S2 Table). For all areas combined, the most abundant fishes, in terms of percentage of overall catches, were Gopher rockfish (*Sebastes carnatus*, 28.1%), Blue rockfish (*S*. *mystinus*, 25.5%), and Black rockfish (*S*. *melanops*, 18.0%).

**Table 1:** Composition of the eleven species most commonly caught during hook-and-line surveys in central California Marine Protected Areas and associated Reference sites from 2007–2013.

Common Name	Scientific Name	Total Catch (%)
Black rockfish	*Sebastes melanops*	18.0
Blue rockfish	*Sebastes mystinus*	25.5
Canary rockfish	*Sebastes pinniger*	1.8
China rockfish	*Sebastes nebulosus*	1.3
Copper rockfish	*Sebastes caurinus*	1.5
Gopher rockfish	*Sebastes carnatus*	28.1
Kelp rockfish	*Sebastes atrovirens*	2.5
Lingcod	*Ophiodon elongatus*	4.0
Olive rockfish	*Sebastes serranoides*	6.6
Vermilion rockfish	*Sebastes miniatus*	4.1
Yellowtail rockfish	*Sebastes flavidus*	2.7

Data from all areas and sites are combined. Total catch equaled 46,853 fishes.

### Initial conditions (2007–2008)

**Fig 3:**
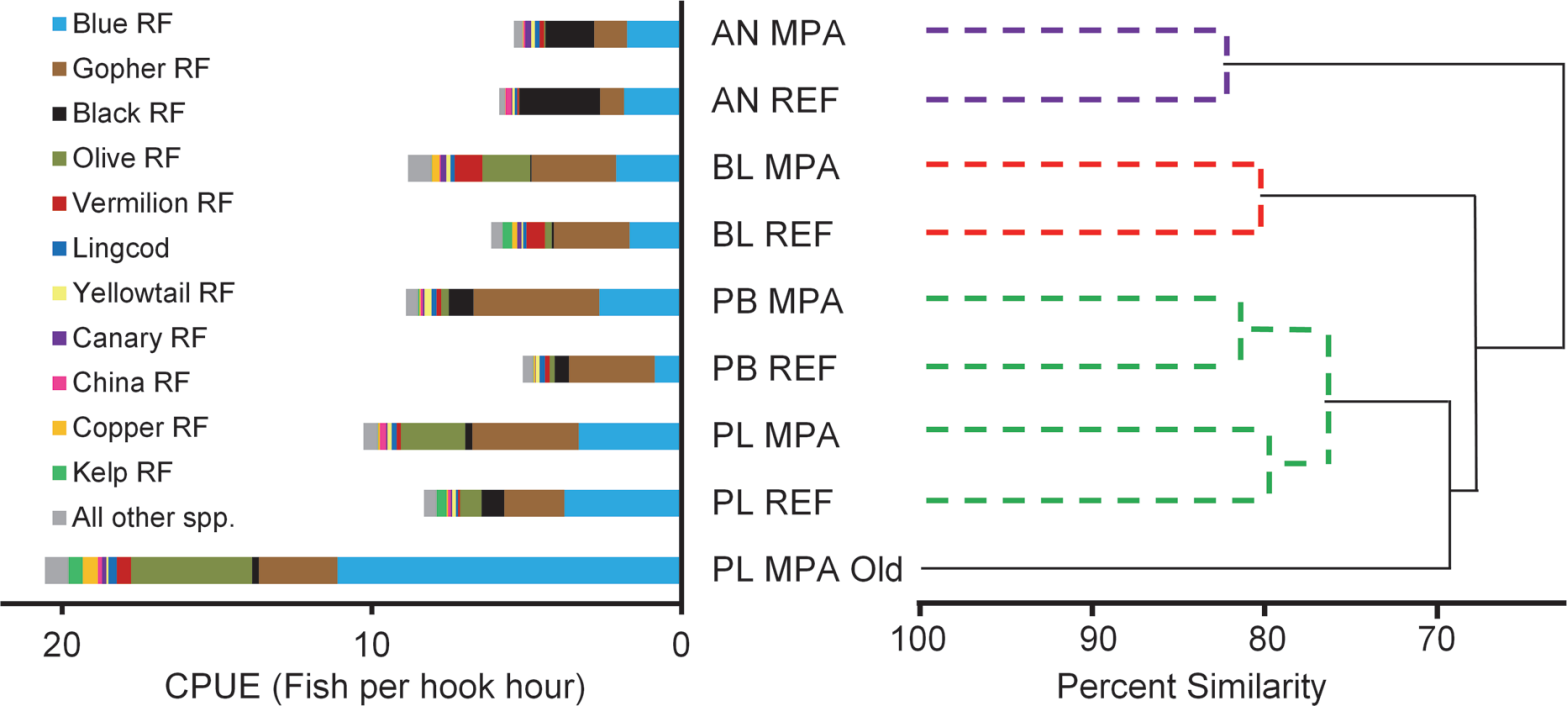
Comparison of species compositions at each marine protected area (MPA) and reference (REF) site. Catch-per-angler-hour (CPUE) data, primarily from rockfishes (RF) caught during the first two years of MPA implementation (2007 and 2008) were incorporated into a Bray-Curtis ordination matrix to compare species similarity among areas. AN: Año Nuevo; BL: Piedras Blancas; PB: Point Buchon; PL: Point Lobos. Dashed lines in the dendrogram indicate statistically significant clusters (p < 0.05).

Bray-Curtis similarity indices indicated that paired MPA and REF sites were > 80% similar in species composition in the first two years after MPA establishment (Fig. 3). Species compositions across geographic areas did not group by latitude but rather by habitat type. Species compositions in the Point Lobos and Point Buchon areas, which contain high relief rock habitats, were more similar to each other than to MPA and REF sites in the Año Nuevo and Piedras Blancas areas, which contained lower-relief rock habitats. Diversity indices were similar for all areas from 2007–2008, but were lower in REF sites than MPAs. The Shannon-Weiner index of diversity ranged from 1.45 to 2.16 in MPAs and from 1.35 to 1.69 in REF sites. Although the habitats occurring in the “Old” portion of the Point Lobos MPA and REF site were similar, the “Old” part of the Point Lobos MPA that was established in 1973 contained a species composition that was unique among areas we surveyed (Fig. 3). Blue rockfish and Olive rockfish (*S*. *serranoides*) comprised the majority (70%) of the catches in the “Old” portion of the Point Lobos MPA in 2007 and 2008. Overall catch rates in the “Old” part of the Point Lobos MPA were significantly higher than in the Point Lobos REF, and the CPUE of seven of the 11 most abundant fishes was significantly higher in the “Old” part of the reserve than in the REF (Table 2). Additionally, mean lengths of nine of the 11 most frequently caught fishes were significantly larger in the “Old” portion of the reserve than in the REF site (Table 2).

**Table 2:** Mean total lengths (cm ± SE) and catch-per-angler-hour (CPUE ± SE) for the eleven most commonly caught fishes inside the "Old” portion of the Point Lobos State Marine Reserve (Old PL, established in 1973) and associated reference (REF) sites.

	Mean Length (cm) ± SE		Mean CPUE ± SE	
	Old PL	REF	Old PL	REF
Common Name	n = 4,334	n = 2,042	n = 420	n = 552
Black rockfish	** 31.8 ± 0.5	29.7 ± 0.2	0.2 ± 0.1	0.8 ± 0.3
Blue rockfish	** 28.8 ± 0.1	26.9 ± 0.2	** 11.3 ± 1.1	3.0 ± 0.5
Canary rockfish	30.6 ± 0.7	29.1 ± 2.0	* 0.1 ± 0.0	0.0 ± 0.0
China rockfish	** 30.2 ± 0.4	26.9 ± 0.5	0.2 ± 0.1	0.1 ± 0.0
Copper rockfish	** 38.6 ± 0.6	31.7 ± 1.7	** 0.4 ± 0.1	0.1 ± 0.0
Gopher rockfish	** 27.5 ± 0.1	26.5 ± 0.1	* 2.6 ± 0.3	1.9 ± 0.2
Kelp rockfish	* 30.9 ± 0.3	30.1 ± 0.3	0.4 ± 0.1	0.3 ± 0.1
Lingcod	* 63.8 ± 1.2	58.6 ± 1.9	** 0.3 ± 0.1	0.1 ± 0.0
Olive rockfish	* 35.5 ± 0.1	34.3 ± 0.3	** 3.4 ± 0.6	0.7 ± 0.1
Vermilion rockfish	* 41.6 ± 0.5	38.0 ± 1.7	** 0.4 ± 0.1	0.2 ± 0.0
Yellowtail rockfish	28.1 ± 1.2	26.4 ± 0.7	0.1 ± 0.0	0.2 ± 0.0
Top 11 Species Combined	-	-	** 19.8 ± 1.7	7.4 ± 0.9

Sample size (n) for length reflects the number of fish measured and for CPUE represents the number of unique sampling cell-days. Statistical significances were obtained from a comparison of means using t-tests and are indicated by asterisks:

*: p < 0.05

**: p < 0.001.

Mixed-model ANOVAs of data collected across all MPAs in 2007 and 2008 showed that, out of 44 possible species-area combinations (11 most commonly caught species*four areas), initial densities and mean lengths differed between MPA and REF sites in all areas studied. Mean lengths of eight species-area combinations were significantly greater (p < 0.05) inside some MPAs than their associated REF sites (Table 3), whereas four species were larger in REF sites. BPUE of 16 species-area combinations was greater inside MPAs than in REF sites (Table 3). Point Lobos contained the highest number of species with greater BPUEs and mean lengths inside the MPA, whereas the Año Nuevo and Piedras Blancas areas contained fewer numbers of species with higher BPUE or mean length inside the MPA than in REF sites.

**Table 3:** Results of mixed-model, repeated measure ANOVAs showing the number of species within each of the four marine protected areas (MPA) that exhibited significantly higher biomass-caught-per-angler-hour (BPUE) and mean lengths in either the MPA or associated reference (REF) sites in 2007 and 2008 (2008 and 2009 in Piedras Blancas).

	BPUE		Mean Length	
Area	MPA	REF	MPA	REF
Año Nuevo	1	2	2	1
Piedras Blancas	3	1	2	2
Point Buchon	5*	-	1	1
Point Lobos	7*	-	3	-
Total	16	3	8	4

These data represent the initial differences between MPA and REF sites at the time of MPA designation in 2007–2008.

* MPAs in which BPUE was significantly different for all species pooled.

### Changes in response variables from 2007–2013

**Fig 4:**
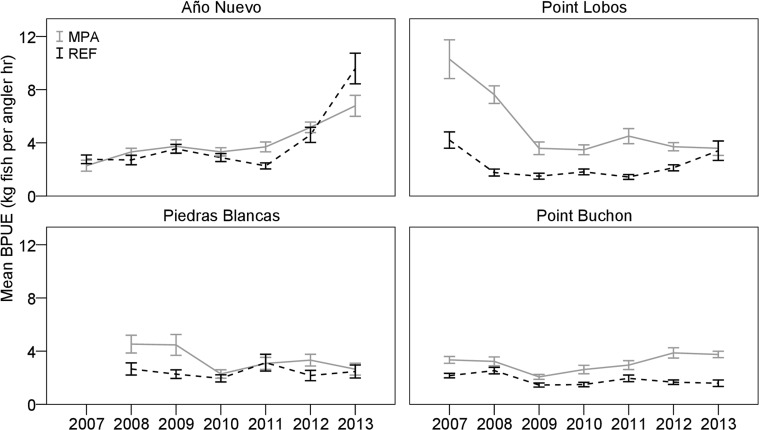
Mean biomass-per-unit-effort (BPUE) of the eleven most commonly caught species from 2007–2013. BPUE is represented as the estimated weight (kg) of fishes caught-per-angler-hour, using two hooks per line. Solid lines illustrate catch rates from marine protected areas (MPA), whereas dashed lines represent values from associated reference (REF) sites. Error bars denote one standard error above and below the mean.

**Fig 5:**
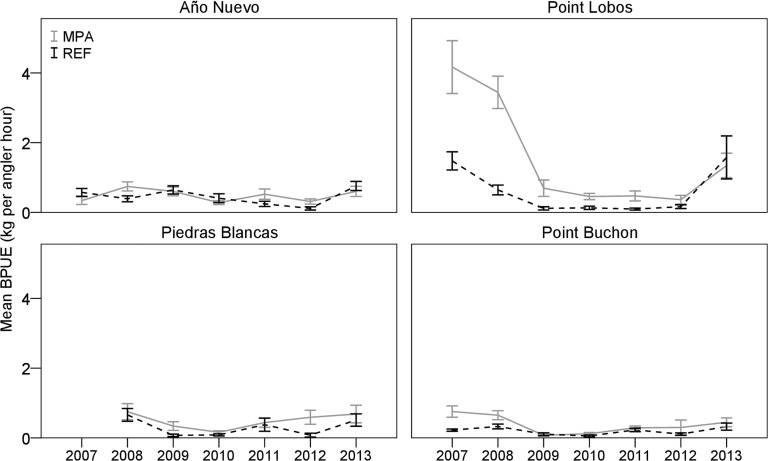
Mean biomass-per-unit-effort (BPUE) of Blue rockfish caught from 2007–2013. BPUE is represented as the estimated weight (kg) of Blue rockfish caught per angler hour, using two hooks per line. Solid lines illustrate catch rates from marine protected areas (MPA), whereas dashed lines represent values from associated reference (REF) sites. Error bars denote one standard error above and below the mean.

**Fig 6:**
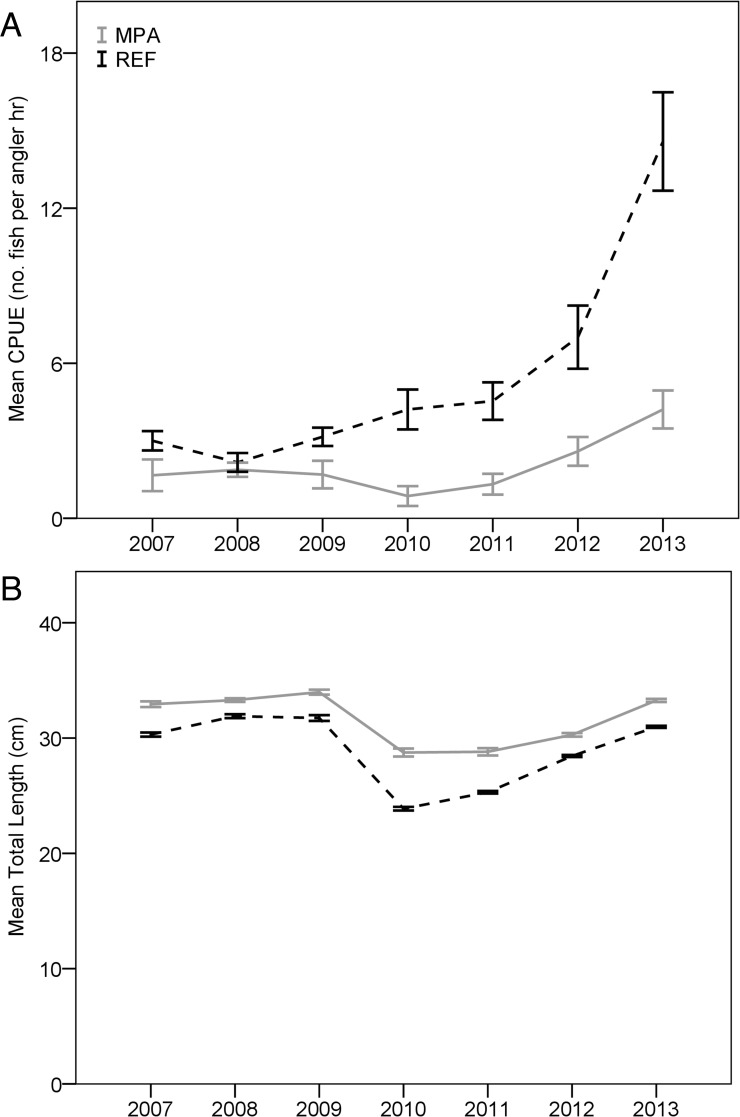
Catch rates (A) and mean lengths (B) of Black rockfish in the Año Nuevo area from 2007–2013. Catch-per-unit-effort (CPUE) is represented as the number of fishes caught-per-angler-hour, using two hooks per line. Solid lines illustrate catch rates or mean lengths from fish caught in the Año Nuevo marine protected area (MPA), whereas dashed lines represent values from associated reference (REF) sites. Error bars denote one standard error above and below the mean.

Diversity indices remained consistent for all areas from 2007–2013, except that the Año Nuevo REF showed a decline in the Shannon-Weiner index of diversity in the 3 years following a strong recruitment of Black rockfish in 2010 (H’ reduced from 1.64 to 0.80). Trajectories of BPUE of all species combined fluctuated over time in MPAs and associated REF sites (Fig. 4). Mean CPUE, BPUE, and lengths of eight of the 11 most frequently caught species changed little over seven years, and the changes that did occur were not in the same direction in all MPAs. Density or lengths of three species, however, did exhibit distinct trends. We observed a decline in the relative abundance of Blue rockfish from 2007–2010 in all MPA and REF sites; the decline was most dramatic in the Point Lobos and Point Buchon MPAs (Fig. 5). A similar pattern was observed with Olive rockfish. Conversely, an episode of strong recruitment of Black rockfish in the Año Nuevo REF site created an increase in BPUE (Fig. 6A) and decrease in mean lengths (Fig. 6B) in both the Año Nuevo MPA and REF sites, although the increase in biomass was greater in the REF site.

Overall, BPUE and mean lengths of each of the top eleven species caught varied by species, area monitored, and by MPA or REF site. No species showed significantly greater BPUE or mean length in all four of the MPAs. Separate runs of the mixed-model ANOVAs for each species at each area using data collected from 2007–2013 provided results that showed that BPUE was significantly higher in MPAs over time for 15 out of 44 possible species-area combinations, and BPUE of three species-area combinations was higher in REF sites (Table 4). Similarly, ten species-area combinations contained mean lengths that were significantly greater over time in MPAs than in REF sites (three species-area combinations contained the opposite pattern). These patterns occurred across all MPAs, as BPUE was greater in MPAs for approximately one-third of the top eleven species in all areas (Table 5). These are similar results as those obtained by the BPUE and mean length model runs for the initial starting conditions.

**Table 4:** Results from mixed-model, repeated-measures ANOVA of individual species showing changes between marine protected areas (MPA) and reference (REF) sites across all years (2007–2013).

	BPUE		Mean length	
Species	MPA	REF	MPA	REF
Black rockfish	-	-	1	-
Blue rockfish	2	-	-	1
Canary rockfish	1	-	1	-
China rockfish	2	1	-	-
Copper rockfish	2	-	2	-
Gopher rockfish	2	1	-	2
Kelp rockfish	-	1	-	-
Lingcod	1	-	1	-
Olive rockfish	-	-	2	-
Vermilion rockfish	2	-	2	-
Yellowtail rockfish	3	-	1	-
Grand Total	15	3	10	3
All species combined	1	-	-	-

Model results describe the number of areas surveyed in which biomass-caught-per-angler-hour (BPUE) or mean lengths of species were significantly higher at either the MPA or REF site when there was no significant interaction between the ‘site’ and ‘year’ term in the model. The range an for individual species is 0–4 areas.

**Table 5:** Results of mixed-model, repeated measure ANOVAs showing the number of species within each of the four marine protected areas (MPA) that exhibited significantly higher biomass-caught-per-unit-effort (BPUE) and mean lengths in either the MPA or reference site (REF) across all survey years (2007–2013).

	BPUE		Mean Length	
Area	MPA	REF	MPA	REF
Año Nuevo	3	1	3	-
Piedras Blancas	4*	2	4	2
Point Buchon	5	-	1	1
Point Lobos	3	-	2	-
Total	15	3	10	3

* MPAs in which BPUE was significantly different for all species combined.

A significant interaction between the ‘year’ and ‘site’ term in some species models showed that trends in biomass and mean length changed over time. For these species-area combinations no clear reserve effect occurred in the time period 2007–2013 (Tables 6, 7). For the BPUE response variable, six of the 44 species-area combinations showed a significant interaction with a more positive slope in the MPA site, and five species-area combinations showed a more positive slope in the reference site. For the mean length response variable there were two species-area combinations showing significant interactions between year and site, one where MPA site displayed a more positive trajectory and one where the REF site showed a more positive trajectory.

**Table 6:** Results from models of biomass-caught-per angler-hour (BPUE) and mean lengths of species when there was a significant interaction between the ‘site’ term (marine protected area (MPA) or reference (REF) site) and the ‘year’ term in the species model.

	BPUE		Mean length	
Species	MPA	REF	MPA	REF
Black rockfish	-	1	-	-
Blue rockfish	-	1	-	-
Canary rockfish	1	-	-	-
China rockfish	-	-	-	-
Copper rockfish	-	1	-	-
Gopher rockfish	-	-	-	-
Kelp rockfish	1	-	-	1
Lingcod	2	-	-	-
Olive rockfish	1	1	1	-
Vermilion rockfish	1	1	-	-
Yellowtail rockfish	-	-	-	-
Grand Total	6	5	1	1
All spp. combined	-	1	-	-

Numbers in each column represent the number of areas with significant differences in BPUE and/or mean length over time (range for an individual species is 0–4 areas).

**Table 7:** Number of species by area which showed a significant interaction between the ‘site’ and ‘year’ term in the model and differences in the overall trend (slope) of biomass-caught-per angler-hour (BPUE) and mean length (P < 0.05).

	BPUE		Mean Length	
Area	MPA	REF	MPA	REF
Año Nuevo	2	1	1	-
Piedras Blancas	-	-	-	1
Point Buchon	4	-	-	-
Point Lobos	-	4*	-	-
Total	6	5	1	1

* significant difference for all spp. combined

An evaluation of changes in trajectories of response variables across multiple MPAs indicated that there were no significant patterns of higher BPUE or mean length in relation to site (MPA or REF) for the time period 2007–2013, except that the mean length of Lingcod (*Ophiodon elongatus*) was greater inside all MPAs after seven years of data collection. Results of model runs for all MPAs as a whole showed substantial variation in slopes of BPUE and mean length among all areas and over time (S3 Table). For example, trends in BPUE of Gopher and China rockfish (*S*. *nebulosus*) varied by area but did not vary significantly over time (S3 Table), whereas Black, Blue, and Olive rockfish and Lingcod BPUE varied across areas and by year (S3 Table).

**Fig 7:**
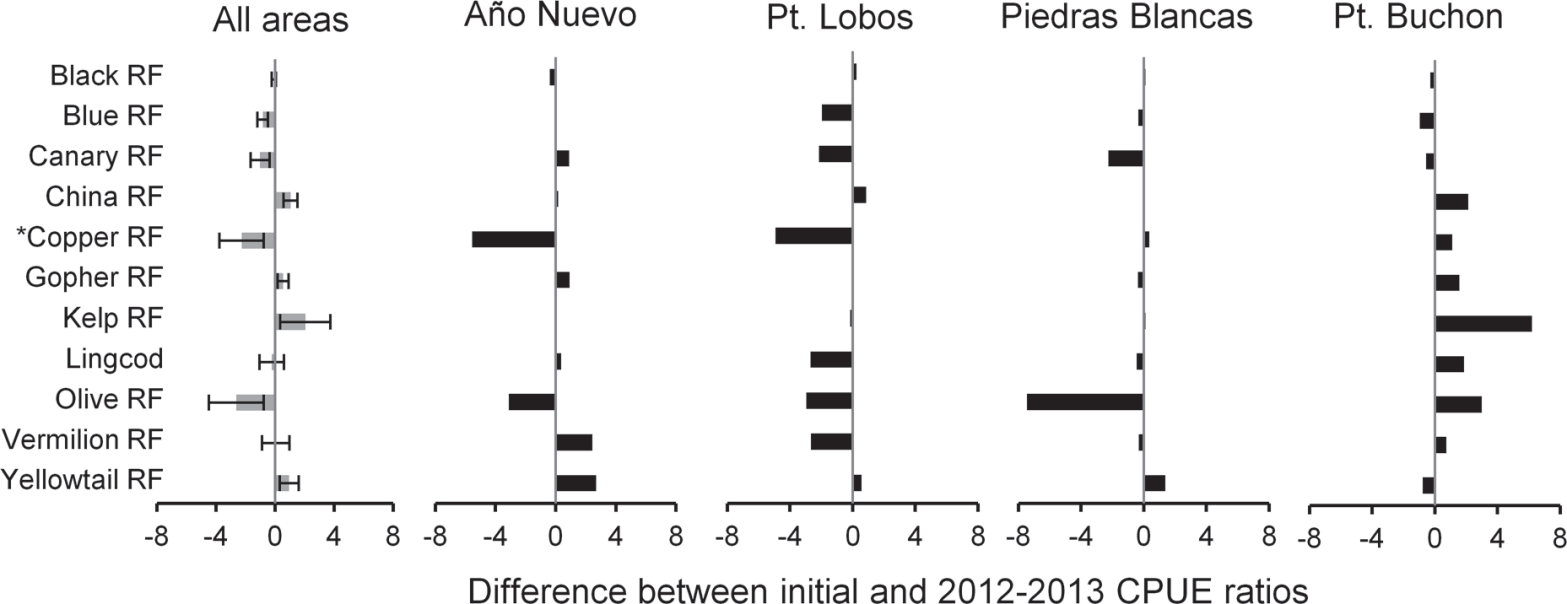
Change in catch-per-angler-hour (CPUE) inside marine protected areas (MPA) and in associated reference (REF) sites. When comparing the change in ratio of CPUE inside MPA and in associated REF sites over time (change in MPA:REF between the initial conditions of 2007–2008 and later conditions in 2012–2013), analyses that use an average across all MPAs can mask large differences in performance of a single MPA. In this plot, positive numbers greater than 0.9 imply more fish in the MPA and an increase over time in the difference between the MPA and REF site. Negative numbers imply that the MPA and REF site became more similar over time or that the ratio switched from having higher CPUE in the MPA initially to higher CPUE in the REF site. Error bars represent mean standard error for all areas. * In the Año Nuevo area, differences shown for Copper rockfish are overestimated due to low numbers of fish caught in the MPA (n = 20) and REF (n = 4) sites.

Throughout the study period, the absolute differences in changes in relative abundance of fishes varied among each species and MPA/REF site. A comparison of the ratio of CPUE inside and outside each MPA during the initial 2 years of implementation (2007–2008) and the last 2 years of data collection (2012–2013) indicated that the relative abundances of some species became more similar in MPA and REF sites, whereas other species showed a divergence in abundances inside and outside MPAs (Fig. 7). For example, the relative abundance of Olive rockfish became more similar between the MPA and REF sites in all areas except at Point Buchon, where the CPUE of Olive rockfish increased by a factor of three inside the MPA. Additionally, the average change in the inside-outside ratio over time for all areas poorly represented the patterns at individual areas. For instance, there was no detectable change in Vermilion rockfish (*S*. *miniatus*) when all years were averaged. However, in the Año Nuevo and Point Buchon areas the ratio of CPUE of Vermilion rockfish inside the MPA relative to the REF increased over time whereas at Piedras Blancas and Point Lobos CPUE became more similar between MPA and REF sites (Fig. 7).

**Fig 8:**
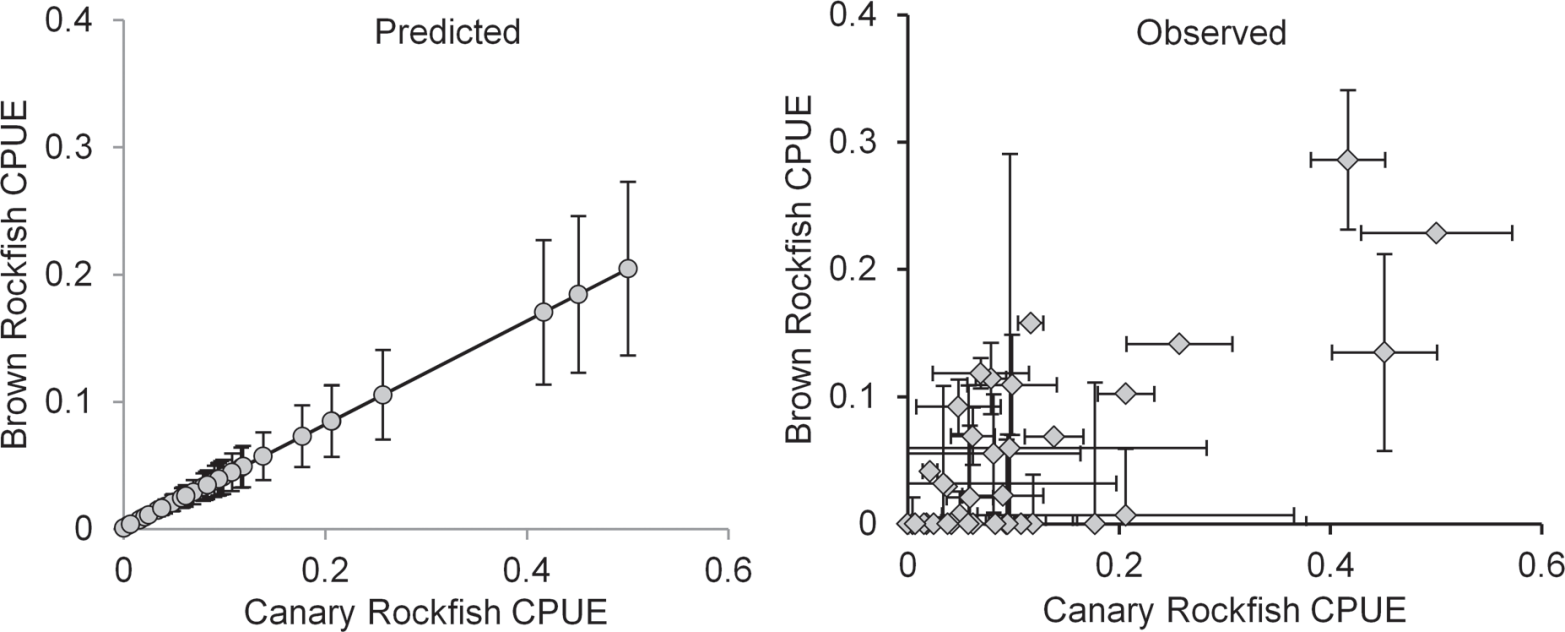
Comparisons of predicted and actual catch rates of Brown and Canary rockfishes. The predicted regression line is based on the relationship between factor score and Canary rockfish catch-per-unit-effort (CPUE).

Factor analyses of all fishes caught yielded positive correlations of CPUE among co-occurring species. However, even the relationship of the two species with the highest loadings in the factor analysis matrix, Brown rockfish (*S*. *auriculatus)* (0.85) and Canary rockfish (*S*. *pinniger*) (0.92), contained high variability in the observed relationship (Fig. 8). This variability was due to the error associated with abundance estimates as well as the error of the slope and intercept of the regression equation. Results such as these reduce confidence in the ability to predict abundances of one species on the basis of another co-occurring species.

**Fig 9:**
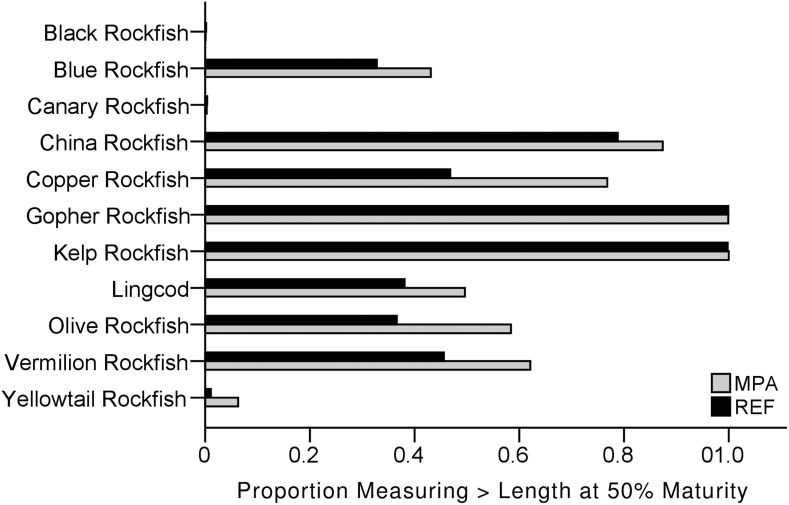
Mature fishes inside protected areas. Proportion of individual fishes for each of the eleven most commonly caught species that were greater than the length at 50% maturity. Data are presented for all marine protected area (MPA) and reference (REF) sites combined from 2007–2013.

Except for Black rockfish in the Año Nuevo area, there was little variation in the frequency distribution of lengths of fishes from 2007–2013. Also, the changes that did occur were similar between the MPA and REF sites for all species. Overall, however, MPAs contained a higher proportion of adult fishes (fish above the length of 50% maturity) than did REF sites. This greater proportion of larger fishes, combined with generally greater biomass in MPAs, resulted in a larger spawning potential biomass in MPAs than in REF sites (Fig. 9).

### Variance explained by spatial temporal and environmental factors

Multiple regression models of catch rates (BPUE) of individual species indicated that spatial factors (cell, site, area and site*area interaction) accounted for the largest amount of explained variance among samples (mean = 20.2%, range 0.6–47.1%). This was followed by factors that described interactions between these spatial factors and time (year*site*area, year*site, year*area; mean = 7.7%, range 1.1–15.4%). Finally, environmental variables describing habitat conditions and weather at the time of fishing accounted for only small amounts of explained variance (mean = 2.1%, range 0.4–3.9%).

### Distance moved (tagged fishes)

As of July 2014, a total of 251 individual tag recaptures have been reported (Table 8). Tagged fishes were recaptured by commercial and recreational hook-and-line fishermen, commercial trap fishermen, SCUBA divers, and during our fishing surveys. Of all the tagged fishes recapture and reported, 71% were recaptured in the same site and grid cell as they were released, and 22% of recaptured fishes were caught within the same site but outside the original grid cell where they were released. Only 18 fish, or 7% of the recaptured fishes, were recaptured beyond the boundaries of the MPA or REF site in which they were released. The mean net distance moved by eight of nine species recaptured was less than half the length of the MPAs we studied.

**Table 8:** Movements of fishes recaptured between 2007 and 2013.

Species	n	Range of Time at Liberty (d)	Minimum Net Distance Moved (km)	Maximum Net Distance Moved (km)	Mean Net Distance Moved (km) ± SD^1^	Mean Net Distance Moved (km) ± SD^2^
Black rockfish	45	4–1,372	0.0	975.8	212.6 **±** 347.3	25.5 **±** 46.2
Blue rockfish	12	1–623	0.0	8.2	1.2 **±** 0.7	2.38 **±** 4.1
Brown rockfish	6	8–1,082	0.1	2.5	0.6 **±** 1.0	0.26 **±** 0.5
Copper rockfish	19	0–1,879	0.0	1.6	0.2 **±** 0.4	0.36 **±** 0.8
Gopher rockfish	59	7–1,744	0.0	42.2	2.7 **±** 7.5	0.53 **±** 0.9
Kelp rockfish	5	271–1,161	0.0	0.2	0.1 **±** 0.1	0.01 **±** 0.1
Lingcod	20	0–1,860	0.0	53.5	3.9 **±** 11.9	12.38 **±** 14.3
Olive rockfish	8	22–431	0.0	7.9	1.1 **±** 2.8	0.36 **±** 1.9
Vermilion rockfish	8	0–1,779	0.0	16.0	3.5 **±** 6.0	0.85 **±** 2.5

The number of fish recaptured (n), time at liberty in days (d), and minimum, maximum and mean distances moved (km ± SD) are reported by species.

^1^ Denotes results from this study.

^2^ Indicates data from the Freiwald [47] literature review for comparison purposes.

The farthest distance moved from release to point of recapture was that of a Black rockfish, released in the Point Lobos REF site, which traveled northward 976 km in 194 days. The shortest distance between release and recapture and the longest time at liberty occurred by a Copper rockfish that was released from the Point Lobos MPA and recaptured > 5 years later, < 0.1 km from the point of release.

## Discussion

It is important to understand the reasons for differences in initial conditions in MPAs and associated REF sites so that changes in trajectories in response variables with time can be properly attributed to reserve effects and not other causes. This is especially true in regions containing fluctuating oceanographic conditions, such as eastern boundary currents and strong upwelling systems. The California Current is characterized by frequent fluctuations in primary production, caused by periodic basin-scale climatic changes [15], and by localized seasonal upwelling events [16], [17]. Even though the timing of fish recruitment is often synchronous along the coast of California [18], [19], changes in primary production can lead to considerable spatial variation in the distribution of zooplankton and ichtyhoplankton [19], [20]. This spatial variability can lead to differences between MPA and REF sites that have more to do with stochastic recruitment than habitat protection. An example of this is the increase in fish densities (and concurrent decrease in mean lengths) in the Año Nuevo REF site that were due to a large recruitment episode of Black rockfish.

Short-term environmental variability can also influence the impression of how MPAs are working. Densities of Blue and Olive rockfish in the beginning of our study decreased dramatically from 2007–2009. We believe the decline was caused by nutrient-poor ocean conditions that occurred in central California from 2004–2006. During those years, the multivariate El Niño–Southern Oscillation (ENSO) index was positive and the Pacific Decadal Oscillation showed a three-year warm period during a decade of cooling [21]. In those years, the California Current was anomalously unproductive [22], [23], causing poor recruitment in rockfishes [19] and large mortalities of juvenile salmon (*Onchorynchus* spp.), seabirds, and marine mammals [24], [25]. Blue and Olive rockfish are relatively short-lived and have small home ranges [26], thus are at risk of starvation when their planktonic prey items are scarce. This is one example of the dangers of relying primarily upon “Expert Opinion” [27] as a primary means of evaluating the status of ocean resources. Without a routine monitoring program, negative changes in MPA response variables could incorrectly be assigned to failure of the MPA when they were actually due to environmental changes at much larger spatial scales.

Initial conditions may affect reserve performance as well. Early MPA modeling studies (e.g., [28–31]) predicted the response of fished populations to protection, independent of habitat quality. More recently, however, Rodwell et al. [32] postulated that recovery of overfished species would occur faster in areas of better habitat, implying that protecting good habitat would be a more effective way to speed recovery of overfished populations. They theorized that by protecting populations in good habitat, fishes would grow larger, faster, and thus reproductive capacity would increase more rapidly than populations in areas with lesser habitat quality. Friedlander et al. [33] and Ortiz and Tissot [34] provided empirical evidence that habitat quality is an important factor in the success of reserves. On a broader scale, after conducting a meta-analysis of 58 studies in 19 marine reserves in southern Europe, Claudet et al. [35] also concluded that habitat quality is a critical factor in determining the effects of marine reserves.

Using the first two years of our surveys as a baseline, it is now clear that at the time of MPA implementation catch rates, biomass, and mean lengths of heavily fished species were greater in newly designated MPAs than in associated REF sites in three of the four areas we studied. The differences in those variables between MPA and REF sites have remained consistent for most species over the seven years of our study. If the initial differences were due primarily to habitat quality, then we may expect population response variables to increase more quickly in the three MPAs that started out with more and larger fishes.

### Changes in response variables over time

MPA modeling studies have indicated that the population demographics of protected species (e.g., rates of natality, mortality, growth, fecundity, etc.) and spatial distribution of habitats and MPAs influence the rate at which species respond to MPA protection (e.g., [36–38]). Empirical results from several MPA monitoring programs around the world, however, have shown that positive reserve effects can occur in short time frames [5], [6], [39]. In many of those studies, short-lived or fast growing species have driven the positive response. In contrast to these empirical results in tropical areas, models developed for temperate regions, and for species such as rockfishes that are long-lived, slow growing, and late to mature, suggest that protection of 10–20 years or more may be needed for reserve effects to become evident [7]. Of the eleven most abundant species in our study, four do not reach the size at 50% maturity until an age of five years [40], and all are considered to have inconsistent rates of recruitment, with large pulses occurring on the scale of a decade [19]. Therefore, the seven-year span of our study is probably too short a time period for large positive reserve effects to accrue because of increased growth and reproductive output of fishes inside MPAs, relative to REF sites. Additionally, because of stringent fishery regulations in California, fishing mortality outside the MPAs in the last decade was probably too low to cause a quick decline in density of fishes outside MPAs.

MPA models indicate that long-lived species groups with infrequent recruitment success, such as rockfishes, will take a longer time to respond positively to protection afforded by MPAs [3], [4], [7]. Although most MPAs we studied exhibited few reserve effects, the part of the Point Lobos MPA that has been closed to fishing since 1973 showed the expected positive responses to protection. Of the eleven most abundant species in the “Old” Point Lobos reserve, nine species were significantly larger and more abundant in the MPA than in the adjacent REF site, even though habitats are very similar. Given that the “Old” reserve was closed to fishing in 1973, and that significant differences in response variables were apparent during the first year of our study, we would expect to see positive reserve effects in other parts of central California within 34 years. This represents an outer boundary of the time for reserve effects to become evident in the temperate waters of central California given a similar suite of species and habitats as Point Lobos. A study by Paddack and Estes [41] provides a logical early boundary. Based on SCUBA surveys of the “Old” Point Lobos reserve conducted 20 years after reserve establishment, they reported significant differences in mean lengths (but not densities) of ten species inside the reserve. Their work corroborates the idea presented by Guidetti and Sala [42] that significant increases in biomass, but not density indicate that the strongest effect of reserve protection is an increase in individual size.

Marine reserves are predicted to experience an increase in diversity after MPA implementation. After seven years of monitoring we saw no trend in diversity indices. It is worth noting, however, that we caught a relatively small number of species. Allen et al. [43] estimated that of about 289 species of marine fishes known in California, more than 70 species inhabit nearshore kelp and rocky habitats in central California. In our study, only eleven species accounted for >96% of the catch. Sample sizes of the other 32 species we caught were too small to provide statistically meaningful estimates of MPA performances. This experience of quantifying only the most common species in an area is typical [44] because there are too many small or cryptic fishes to accurately quantify species richness using non-destructive techniques. Our data indicate that using species richness or diversity as a response variable for change detection is less meaningful than using changes in densities or sizes of fishes. This is not a major issue if the reserve goal is to protect large, fished species, because Claudet et al. [5] suggested that the most sensitive MPA performance metrics are related to large species and species targeted by fishing.

In addition to understanding how habitats and other environmental factors affect the relative abundance of species, it is important to understand how management actions affect fish populations outside MPAs. As a result of stringent management actions, populations of many eastern Pacific rockfishes have increased since 2002 [45]. Increased population sizes, combined with improved ocean conditions for rockfishes, have resulted in more frequent episodes of successful recruitment in central California [19] and will lead to increased fish abundances both inside and outside MPAs. Although successful rockfish recruitment has been more frequent in the past decade, the spatial and temporal variability in recruitment of California fishes is extremely high. Ralston et al. [19] reported that juvenile abundances of ten species collected in larval form varied by factors of 12–47 over a 28-year period.

### Spillover

Many of the species we caught and tagged are known to be residential [26], [46], [47] and tag returns from our study indicated little spillover from MPAs. The small amount of tagged fish that were caught outside MPAs provides evidence that central California MPAs are large enough to protect the fishes we studied. A notable exception is that 21 Black Rockfishes tagged in central California have been recaptured > 350 km north of release locations, suggesting that the mobile species venturing outside MPAs in central California could provide spillover benefits to fisheries. Although we saw little evidence of fish emigration from reserves, we did not explicitly design our studies to evaluate rates or directions of spillover. Harmelin-Vivien et al. [48] identified the need for sampling along a gradient to identify spillover effects, and Guidetti and Sala [42] and Stobart et al. [49] identified the need to use multiple community metrics in order to evaluate changes outside MPAs. Also, we did not evaluate the effect of “fishing the line”, which Kellner et al. [50] suggested could affect interpretations of reserve protection and spillover.

### MPA networks

Several models have been developed that describe potential MPA network benefits (e.g., [37], [44], [51]). Inherent in the presumed benefits of a network are the assumptions that populations in each MPA persist and are connected by transport of larval and/or juvenile stages of species among MPAs in the network. Adaptive management of a MPA network may be difficult given that existing networks usually have been developed by distributing MPAs across a geographic area without the empirical or theoretical information needed to provide an estimate of expected benefits [52]. Network goals have rarely been articulated and, except for the work described by Hamilton et al. [53] and Harrison et al. [54], have not been evaluated.

Although we did not evaluate juvenile or larval connectivity of fishes among MPAs, we did review our data to determine if the information we collected from any one MPA could be used to evaluate a collection of MPAs. Our rationale was that a collection of MPAs in a region might be viewed as connected if all MPAs responded similarly over time. The fluctuations in trajectories of response variables among species and MPA/REF pairs were synchronous in three of the four MPAs we monitored. This result would indicate that one MPA might be an indicator of central California network response; however, the large recruitment episode of Black rockfish at Año Nuevo MPA and REF sites demonstrates why using just one MPA as an indicator for an entire network could be problematic.

To interpret changes in response variables at a regional scale, it is important to understand the contribution of each habitat type to the observed changes. A large change in one MPA (relative to its REF site) can provide a biased view of how a network has responded, if response ratios are pooled or averaged across species or MPAs. We suggest that rather than averaging response variables across all MPAs in a network, it is more appropriate to follow trajectories of response variables in each MPA. Lester et al. [6] implied this same idea when they suggested that the lack of a reserve effect in some meta-analyses might be due to the aggregation of data over multiple species. This also supports the ideas of White et al. [38] who reported that model results showed wide variation in performance of different species within the same reserve networks, and suggested avoiding the use of multi-species averages. They also counseled against the use of a single MPA for assessing MPA network performance.

Resource managers concerned about cost-effectiveness of monitoring often search for a single species to use as an indicator of MPA performance. Our results showed that several species could be used as indicators of general trends. However, the errors associated with the regressions were high, indicating that it would be difficult to predict the amount of change in an uncommon species based on the magnitude of change from an abundant species. Claudet et al. [5] reported similar results in a study of Mediterranean MPAs and suggested that community-level metrics may provide a more robust understanding of MPA effects than an indicator species. Thus, indicator species might be useful for getting a general idea of MPA performance, but not useful for estimating the magnitude of MPA or network responses.

## Conclusions

The MPA network established in central California contains long-lived, slow growing species that are adapted to a highly variable eastern boundary current ecosystem. Our fishery-independent surveys in central California MPAs showed that upon implementation, most MPAs contained slightly more and larger fishes than associated REF sites, probably due to differences in habitat. Most differences between MPA and REF sites did not greatly change over the seven years of our study indicating that reserve benefits likely will be slow to accumulate in central California’s ecosystem. SCUBA surveys after 20 years of protection in the older portion of the Point Lobos Marine Reserve suggested little change in fish populations; however, our surveys conducted there after 34 years of protection showed significantly more abundant and larger fishes in the MPA than the associated REF site. This indicates that we can expect reserve benefits to accrue in the temperate California Current ecosystem, but that 20 years or more may be needed to detect significant changes in response variables that are attributable to MPAs. Because of the high spatial and temporal variability of recruitment patterns, long-term monitoring will be needed to quantify responses of fishes in the diverse set of habitats in central California waters. Also, the use of one species or MPA as an indicator is unlikely to provide sufficient resolution to accurately describe network performance.

We suggest that an effective way to monitor MPAs is to develop collaborative projects with recreational anglers and to conduct standardized fishery-independent catch and release fishing surveys in MPA and REF sites. The data derived from science-based collaborative fishing projects are sufficiently robust to detect significant differences in fish abundance and sizes. In addition to the scientific credibility of the data, fishermen are more likely to accept the veracity of the information because they or their peers have been involved in project development and collection of the data. In this way, collaborative fisheries research can greatly contribute to the realization of community-based co-management of marine protected areas [55]. Additionally, the fishery-independent data collected while monitoring MPAs can be used in traditional stock assessments and data-poor fishery models to manage nearshore fisheries (e.g., [56], [57]).

## Supporting Information

S1 Table Composition of fishes caught during hook-and-line surveys from 2007–2013. (DOCX)

S2 Table Composition of fishes caught by area during hook-and-line surveys from 2007–2013. (DOCX)

S3 Table Results of network-wide biomass-caught-per angler-hour (BPUE) and mean length species models. Results indicate interaction between the site, area, and year variables in the model.
(DOCX)
