# Supplementary material for: Variation in Responses of Fishes across Multiple Reserves within a Network of Marine Protected Areas in Temperate Waters
Source: PLoS One. 2015 Mar 11;10(3):e0118502. doi: 10.1371/journal.pone.0118502 (PMC4356516; doi:10.1371/journal.pone.0118502)
Supplement: S1 Table — (DOCX) [file pone.0118502.s001.docx]

**Table S1**. Composition of fishes caught during hook-and-line surveys from 2007–2013, all areas and sites combined. Asterisks (*) indicate values < 0.1% of the total catch (n = 46,853).

| Common Name | Scientific Name | Percent of Total Catch (%) |
| --- | --- | --- |
| Black-and-Yellow rockfish | *Sebastes chrysomelas* | 0.3 |
| Black rockfish | *Sebastes melanops* | 18.0 |
| Black Surfperch | *Embiotoca jacksonii* | * |
| Blue rockfish | *Sebastes mystinus* | 25.5 |
| Bocaccio | *Sebastes paucispinis* | * |
| Brown rockfish | *Sebastes auriculatus* | 0.8 |
| Bull Sculpin | *Enophrys taurina* | * |
| Cabezon | *Scorpaenichthys marmoratus* | 0.4 |
| Calico rockfish | *Sebastes dalli* | * |
| California Halibut | *Paralichthys californicus* | * |
| California Lizardfish | *Synodus lucioceps* | 0.1 |
| Canary rockfish | *Sebastes pinniger* | 1.8 |
| China rockfish | *Sebastes nebulosus* | 1.3 |
| Copper rockfish | *Sebastes caurinus* | 1.5 |
| Gopher rockfish | *Sebastes carnatus* | 28.1 |
| Grass rockfish | *Sebastes rastrelliger* | * |
| Jack Mackerel, Pacific | *Trachurus symmetricus* | 0.1 |
| Kelp Greenling | *Hexagrammos decagrammus* | 0.7 |
| Kelp rockfish | *Sebastes atrovirens* | 2.5 |
| Lingcod | *Ophiodon elongates* | 4.0 |
| Ocean Whitefish | *Caulolatilus princeps* | * |
| Olive rockfish | *Sebastes serranoides* | 6.6 |
| Pacific Bonito | *Sarda chiliensis lineolata* | * |
| Pacific Sardine | *Sardinops sagax* | * |
| Painted Greenling | *Oxylebius pictus* | * |
| Petrale Sole | *Eopsetta jordani* | * |
| Pile Perch | *Rhacochilus vacca* | * |
| Rock Greenling | *Hexagrammos lagocephalus* | * |
| Rock Sole | *Lepidopsetta bilineata* | 0.1 |
| Rosy rockfish | *Sebastes rosaceus* | 0.6 |
| Sanddab, Pacific and Speckled | *Citharichthys spp.* | 0.4 |
| Sand Sole | *Psettichthys melanostictus* | * |
| Silver Salmon | *Oncorhynchus kisutch* | * |
| Silversides | Family Atherinopsidae | * |
| Staghorn Sculpin | *Leptocottus armatus* | * |
| Starry rockfish | *Sebastes constellatus* | 0.1 |
| Striped Surfperch | *Embiotoca lateralis* | * |
| Treefish | *Sebastes serriceps* | 0.2 |
| Tubesnout | *Aulorhynchus flavidus* | * |
| Vermilion rockfish | *Sebastes miniatus* | 4.1 |
| White Croaker | *Genyonemus lineatus* | * |
| Wolf Eel | *Anarrhichthys ocellatus* | * |
| Yellowtail rockfish | *Sebastes flavidus* | 2.7 |
