# Supplementary material for: Variation in Responses of Fishes across Multiple Reserves within a Network of Marine Protected Areas in Temperate Waters
Source: PLoS One. 2015 Mar 11;10(3):e0118502. doi: 10.1371/journal.pone.0118502 (PMC4356516; doi:10.1371/journal.pone.0118502)
Supplement: S2 Table — (DOCX) [file pone.0118502.s002.docx]

**Table S2**. Composition of fishes caught from 2007–2013 (^ 2008–2013), by area. Asterisks (*) indicate values < 0.1% of the total catch for each MPA/REF pair.

|  | | Percent of Total Catch, by Area (%) | | | | | | | | | |
| --- | --- | --- | --- | --- | --- | --- | --- | --- | --- | --- | --- |
|  | | Año  Nuevo | | Point  Lobos | | Piedras  Blancas^^^ | | Point  Buchon | | All  Areas | |
| Common Name | | n = 14,933 | | n = 13,891 | | n = 8,796 | | n = 9,233 | | n = 46,853 | |
| Black-and-Yellow rockfish | | 0.4 | | * | | 0.1 | | 0.6 | | 0.3 | |
| Black rockfish | | 45.4 | | 3.2 | | 2.4 | | 10.8 | | 18.0 | |
| Black Surfperch | | - | | - | | * | | - | | * | |
| Blue rockfish | | 21.3 | | 38.2 | | 21.0 | | 17.5 | | 25.5 | |
| Bocaccio | | - | | * | | 0.1 | | * | | * | |
| Brown rockfish | | 1.2 | | * | | 2.0 | | 0.2 | | 0.8 | |
| Bull Sculpin | | - | | - | | * | | * | | * | |
| Cabezon | | 0.5 | | 0.2 | | 0.2 | | 0.6 | | 0.4 | |
| Calico rockfish | | - | | - | | * | | * | | * | |
| California Halibut | | - | | - | | * | | - | | * | |
| California Lizardfish | | - | | * | | 0.3 | | 0.2 | | 0.1 | |
| Canary rockfish | | 3.0 | | 0.9 | | 1.8 | | 1.4 | | 1.8 | |
| China rockfish | | 1.8 | | 1.6 | | 0.5 | | 0.6 | | 1.3 | |
| Copper rockfish | | 0.3 | | 2.0 | | 3.6 | | 0.7 | | 1.5 | |
| Gopher rockfish | | 14.6 | | 26.5 | | 35.0 | | 45.8 | | 28.1 | |
| Grass rockfish | | 0.1 | | * | | - | | - | | * | |
| Jack Mackerel | | * | | 0.1 | | 0.1 | | * | | 0.1 | |
| Kelp Greenling | | 1.3 | | 0.4 | | 0.5 | | 0.5 | | 0.7 | |
| Kelp rockfish | | * | | 4.4 | | 4.0 | | 2.0 | | 2.5 | |
| Lingcod | | 4.1 | | 3.5 | | 3.1 | | 5.4 | | 4.0 | |
| Ocean Whitefish | | - | | 0.1 | | 0.1 | | - | | * | |
| Olive rockfish | | 0.3 | | 12.5 | | 11.1 | | 3.8 | | 6.6 | |
| Pacific Bonito | | - | | - | | * | | - | | * | |
| Pacific Sardine | | * | | - | | - | | - | | * | |
| Painted Greenling | | * | | - | | - | | * | | * | |
| Petrale Sole | | * | | - | | - | | - | | * | |
| Pile Perch | | * | | - | | - | | - | | * | |
| Rock Greenling | | * | | * | | - | | - | | * | |
| Rock Sole | | * | | 0.1 | | 0.1 | | 0.1 | | 0.1 | |
| Rosy rockfish | | 0.1 | | 1.1 | | 0.2 | | 1.0 | | 0.6 | |
| Sanddab spp. | | * | | 0.1 | | 1.3 | | 0.5 | | 0.4 | |
| Sand Sole | | * | | - | | - | | - | | * | |
| Silver Salmon | | * | | - | | - | | - | | * | |
| Silverside spp. | | * | | - | | * | | * | | * | |
| Staghorn Sculpin | | - | | * | | - | | - | | * | |
| Starry rockfish | | - | | 0.1 | | 0.2 | | 0.3 | | 0.1 | |
| Striped Surfperch | | * | | * | | - | | * | | * | |
| Treefish | | - | | 0.1 | | 0.2 | | 1.0 | | 0.2 | |
| Tubesnout | | - | | - | | - | | * | | * | |
| Vermilion rockfish | | 3.2 | | 2.1 | | 9.1 | | 3.6 | | 4.1 | |
| White Croaker | | 0.1 | | - | | - | | - | | * | |
| Wolf Eel | | - | | - | | - | | * | | * | |
| Yellowtail rockfish | | 2.3 | | 2.5 | | 3.1 | | 3.3 | | 2.7 | |
| Total Number Species | | 30 | | 28 | | 30 | | 30 | | 43 | |
| Total rockfish Species | | 14 | | 17 | | 17 | | 17 | | 18 | |
