# Supplementary material for: Variation in Responses of Fishes across Multiple Reserves within a Network of Marine Protected Areas in Temperate Waters
Source: PLoS One. 2015 Mar 11;10(3):e0118502. doi: 10.1371/journal.pone.0118502 (PMC4356516; doi:10.1371/journal.pone.0118502)
Supplement: S3 Table — Results indicate interaction between the site, area, and year variables in the model. (DOCX) [file pone.0118502.s003.docx]

**Table S3**. Results of network-wide biomass-caught-per angler-hour (BPUE) and mean length species models with significant interaction of explanatory variables. Independent variables in the models were: site (MPA, REF), year (2007–2013), area (Año Nuevo, Point Lobos, Piedras Blancas, Point Buchon).

|  | BPUE | | Mean Length | |
| --- | --- | --- | --- | --- |
| Species | site*area | year*site*area | site*area | year*site*area |
| Black rockfish | - | ** | *** | - |
| Blue rockfish | - | ** | - | - |
| Canary rockfish | - | - | - | - |
| China rockfish | *** | - | - | - |
| Copper rockfish | - | - | ** | - |
| Gopher rockfish | *** | - | - | *** |
| Kelp rockfish | - | - | - | - |
| Lingcod | - | *** | - | - |
| Olive rockfish | - | *** | - | * |
| Vermilion rockfish | - | - | *** | - |
| Yellowtail rockfish | - | - | * | - |
| Total | 2 | 4 | 4 | 2 |
| All species combined | - | *** | - | - |

*p < 0.05, **p < 0.01, ***p < 0.005
